# Comparative Evaluation of Pain Perception Between Needleless Jet Infiltration Anaesthesia and Conventional Needle Anaesthesia for Maxillary Primary Molars in Children: A Systematic Review and Meta-Analysis

**DOI:** 10.7759/cureus.100909

**Published:** 2026-01-06

**Authors:** Utkarsha Kadam, Ritesh Kalaskar, Ashita R Kalaskar, Nidhi Sharma, Snehal Jagtap

**Affiliations:** 1 Department of Paediatric and Preventive Dentistry, Government Dental College and Hospital, Nagpur, Nagpur, IND; 2 Department of Oral Medicine and Radiology, Government Dental College and Hospital, Nagpur, Nagpur, IND

**Keywords:** local anaesthesia, needleless jet injection, pain, pulpectomy, systematic reviews

## Abstract

The use of a syringe and needle for delivering local anaesthesia is known to provoke anxiety and apprehension in patients even before dental treatment begins. To overcome anxiety associated with needles, needleless jet infiltration anaesthesia using the INJEX® system (Pharma AG, Berlin, Germany) has been investigated. The present systematic review and meta-analysis aim to evaluate differences in pain perception among children administered INJEX and conventional infiltration anaesthesia.

A comprehensive literature search was conducted across multiple electronic databases for studies published up to December 31, 2023. Two reviewers independently screened and assessed the articles, and six studies were finally included based on predefined inclusion and exclusion criteria. The risk of bias in the selected randomised controlled trials was evaluated using the Risk of Bias 2 (RoB 2) tool.

Findings of this meta-analysis suggest that pain experienced during injection and treatment with INJEX was not significantly different from that associated with conventional infiltration anaesthesia. In paediatric dentistry, needleless jet injection could represent a preferable option to conventional local anaesthetic delivery, owing to its higher child acceptance and reduced postoperative pain.

Incorporating needleless jet injection (INJEX) into pediatric dental practice can improve patient acceptance by lowering pain and anxiety, ultimately leading to a more positive treatment experience. It offers a child-friendly alternative to conventional needle infiltration, enhancing overall clinical efficiency and patient satisfaction.

## Introduction and background

The introduction of anaesthesia has been a major advancement in dentistry, making routine and invasive procedures more comfortable for patients [1]. In paediatric dentistry, achieving effective pain control is particularly important, as early negative experiences can reinforce dental fear and hinder long-term cooperation [2]. Although local anaesthesia (LA) reliably blocks pain transmission through inhibition of sodium ion influx in neural fibres, the traditional needle-based method of delivering LA remains one of the most anxiety-provoking aspects of dental care for children [3]. Needle phobia, anticipation of pain, and visual exposure to the needle can trigger behavioural distress and reduced cooperation, often exceeding the discomfort caused by the injection itself [4,5].

To overcome these limitations, several alternative anaesthetic delivery systems have been developed, including computer-assisted devices, controlled-rate injectors, and needle-free jet injectors [6]. Among these, needle-free jet systems have gained attention for their potential to minimise pain and anxiety by eliminating the use of a needle. These devices deliver a small volume of solution (typically 0.2-0.3 mL) through a micro-orifice at high pressure within milliseconds, allowing penetration into soft tissue without a needle. However, their clinical performance may be influenced by factors such as audible discharge, abrupt pressure sensation, and variability in penetration depth, which can affect acceptance in children.

Needle-free jet injectors are devices that employ mechanical (spring- or gas-driven) mechanisms to generate a high-velocity liquid jet capable of crossing the mucosal or skin surface without a needle; these devices represent an alternative to hypodermic injections for transdermal or mucosal drug delivery [7]. Its design aims to reduce both pain and needle-related fear, making it a potentially child-friendly alternative to conventional infiltration anaesthesia. Yet, despite increasing clinical interest, evidence supporting its effectiveness and acceptance in paediatric patients remains inconsistent. Arapostathis et al. reported limited data on children’s acceptance of the needleless method [8], while Geenen et al. observed that some children experienced discomfort related to the sudden noise and pressure generated by the INJEX [9].

These mixed observations highlight a significant gap in the literature: the absence of high-quality, paediatric-focused trials specifically evaluating pain perception in commonly treated sites such as the maxillary primary molars, where infiltration anaesthesia is routinely administered. To address this gap, a systematic review was conducted to compare pain perception during injection, measured using validated scales, between needle-less jet infiltration anaesthesia using the INJEX® system (Pharma AG, Berlin, Germany) and conventional infiltration anaesthesia in children.

## Review

Methods

Protocol and Registration

This systematic review and meta-analysis were conducted in accordance with the Preferred Reporting Items for Systematic Reviews and Meta-Analyses (PRISMA) 2020 guidelines. The review protocol was prospectively registered in PROSPERO (CRD42024528717).

Eligibility Criteria

Studies were eligible if they met the following PICOS criteria: Population (P): children aged 3-12 years undergoing dental procedures involving maxillary primary molars; Intervention (I): needleless jet infiltration anaesthesia using the INJEX system; Comparison (C): conventional needle infiltration anaesthesia; Outcome (O): pain perception, assessed using validated measures such as the Visual Analogue Scale (VAS), Wong-Baker Faces Pain Scale, Face, Legs, Activity, Cry, and Consolability (FLACC) scale, or Faces Pain Scale-Revised (FPS-R); and Study design (S): randomised controlled trials (RCTs), including parallel or split-mouth designs, published in English.

Exclusion criteria included observational studies, non-randomised trials, case reports, in vitro or animal studies, adult samples, and studies without extractable pain data.

Information Sources and Search Strategy

A comprehensive electronic search was performed in PubMed, Scopus, Web of Science, Cochrane CENTRAL, and Google Scholar (for grey literature) from inception to December 2023. Search terms combined MeSH and free-text keywords: “needleless anaesthesia,” “jet injection,” “INJEX,” “paediatric dentistry,” “maxillary primary molar,” “local anaesthesia,” and “pain perception.”

The complete search strategies used for each database are presented in Table 1.

**Table 1:** Summary of the search strategy.

Database	Search Strategy	Findings
PubMed	#1(("Dental phobia"( Title/Abstract))) OR ("Local anesthesia"(Title/Abstract))) OR (("Maxillary infiltration"(Title/Abstract))) OR (“Painless dentistry” (Title/Abstract))) #2 ("Needle less anesthesia"(Title/Abstract))) OR ("pressure anesthesia" (Title/Abstract))) OR ("needle free anesthesia" (Title/Abstract))) OR ("jet injection"(Title/Abstract))) OR ("INJEX"(Title/Abstract)) AND ("Needle anesthesia"(Title/Abstract))) OR ("dental phobia" (Title/Abstract))) OR ("needle phobia" (Title/Abstract))) OR ("local anesthesia"(Title/Abstract))) #3 (“pain”)( Title/Abstract))) OR (“efficacy” (Title/Abstract))) #1 AND #2 AND #3	446
Google scholar	Local anesthesia Maxillary primary molar, Needle Anesthesia Infiltration Needle less Anesthesia	463
Cochrane	#1 MeSH descriptor: "local anesthesia" explode all trees	226
	#2 (("Dental phobia"Mesh)) OR ("Local anesthesia"Mesh)) (("Maxillary infiltration"Mesh)) OR (“Painless dentistry” Title/Abstract)) :ti,ab,kw (Word variations have been searched)	
	#1 or #2	
	#3 MeSH descriptor: (needle less anesthesia) explode all trees	
	#4 (("Needle less anesthesia"(Title/Abstract))) OR ("pressure anesthesia" (Title/Abstract))) OR ("needle free anesthesia" (Title/Abstract))) OR ("jet injection"(Title/Abstract))) OR ("INJEX"(Title/Abstract))) :ti,ab,kw (Word variations have been searched)	
	#3 or #4	
	#5 MeSH descriptor: [needle anesthesia] explode all trees	
	#6 ("Needle anesthesia"[Title/Abstract])) OR ("dental phobia" [Title/Abstract])) OR ("needle phobia" [Title/Abstract])) OR ("local anesthesia"[Title/Abstract])) :ti,ab,kw (Word variations have been searched)	
	#5 or #6	

Study Selection

After deduplication, titles and abstracts were screened independently by two reviewers. Full-text articles were then assessed against eligibility criteria. Disagreements were resolved through discussion or consultation with a third reviewer.

Studies comparing the impact of needleless jet infiltration with traditional needle infiltration on children's perceptions of pain and anxiety undergoing maxillary molar infiltration during dental operations were included in the inclusion criteria. Only English-language publications of RCTs, study design (parallel and split-mouth RCTs), acceptable pain assessment tools, minimum reporting requirements, age limits, and intervention/comparator specifications were deemed eligible. Review papers, case series, in vitro or animal research, studies that were only available as abstracts without the complete text, and studies assessing alternative filling techniques and quasi-experimental designs were excluded.

Data extraction covered the following variables: author details, country, year of publication, study title, study design, sample size, participants’ age group and gender, outcomes assessed, key findings, and other relevant information. Two reviewers independently retrieved and summarised data from the included studies. Methodological issues such as randomisation, allocation concealment, and blinding were also assessed.

The Cochrane Collaboration's RCT tool was used to assess the risk of bias (RoB) in each study. This method was used to evaluate the RoB for each included study in the following areas: blinding of participants, allocation concealment, random sequence creation, personnel and result assessment, selective reporting, and other possible sources of bias. Each study's overall quality and validity were then classed as having a low, unclear, or high RoB.

The general characteristics of the included studies are shown in Table 2.

**Table 2:** General characteristics of included studies.

S. No.	Author, Year	Study Sample	Intervention	Comparator	Primary Outcome Pain Scores		Other Outcomes		Conclusion
					Needleless Anaesthesia	Needle Anaesthesia	Needleless Anaesthesia	Needle Anaesthesia	
1	El-Dien Mohamed et al., 2023 [10]	Total sample: 56 maxillary primary molars; Males: 41.1% Females: 58.9% Mean age: 6.84 ± 0.69 years Group I (intervention group): 28 maxillary primary molars Group II (control group): 28 maxillary primary molars	The jet injector was used to deliver 4% articaine with 1:100,000 epinephrine as the injectable local anaesthetic solution.	Buccal infiltration was performed using a conventional syringe with a 30-gauge short needle.	After injection: 2.38 ± 1.96 After pulpotomy: 1.29 ± 1.36 After stainless steel crown preparation: 2.72 ± 2.07	After injection: 2.79 ± 2.33 After pulpotomy: 1.43 ± 1.71 After stainless steel crown preparation: 3.56 ± 3.00	Sound, Eye, Motor (SEM) scale: After injection: 5.21 ± 1.66 After pulpotomy: 4.18 ± 1.85 After stainless steel crown preparation: 3.84 ± 1.34 Adverse effects: Bleeding at the injection site: 7.1% Anaesthetic success: Overall success of anaesthesia: 89.3% Success of local anaesthesia during stainless steel crown placement: 100%	Sound, Eye, Motor scale After the injection -6.11±2.33 After the pulpotomy -4.46±1.90 After the stainless steel crown preparation- 5.59±2.04 Adverse effect - Bleeding in injection site-10.7% Hematoma at injection site-10.7% Bad taste-7.1% Success of anaesthesia 92.9% success of local anaesthesia during the stainless steel crown-92.9%	Buccal infiltration was performed using a conventional syringe with a 30-gauge short needle.
2	Altan et al., 2021 [11]	Total sample: 56 maxillary primary molars Males: 25; Females: 31 Mean age: 6.36 ± 1.06 years Group I (intervention group): 28 maxillary primary molars Group II (control group): 28 maxillary primary molars	A needle-free system with 2% lidocaine and 1:80,000 epinephrine (lidocaine, Colombia) was injected using the Comfort-In™ system. Approximately 0.2 mL of the anaesthetic solution was deposited for filling and 0.3 mL for pulpotomy treatment, delivered to the cutaneous/subcutaneous tissue at a pressure of 2000 psi in less than 2 seconds.	Buccal infiltration was performed using a conventional syringe with a 27-gauge short needle.	Filling: Induction: 2 (0-4) Treatment: 2 (0-4) Post-treatment: 0 (0-2) Pulpotomy: Induction: 2 (0-4) Treatment: 4 (0-6) Post-treatment: 0 (0-2)	Filling: Induction: 6 (6-8) Treatment: 0 (0-4) Post-treatment: 0 (0-2) Pulpotomy: Induction: 6 (2-10) Treatment: 2 (2-4) Post-treatment: 2 (0-4)	-	-	Buccal infiltration was performed using a conventional syringe with a 27-gauge short needle.
3	Ellatif, 2018 [12]	Total sample: 30 children Males: 17; Females: 13 Mean age: 9 years Group I (intervention group): 15 maxillary primary molars Group II (control group): 15 maxillary primary molars	2% lidocaine with 1:80,000 epinephrine (lidocaine, Colombia) was injected using a needle-free system.	Buccal infiltration was performed using a conventional syringe with a 27-gauge short needle.	Faces Pain Scale-Revised: 2.73 ± 0.96	Faces Pain Scale-Revised: 1.13 ± 0.92	-	-	Buccal infiltration was performed using a conventional syringe with a 27-gauge short needle.
4	Belevcikli et al., 2023 [13]	Total sample: 94 children (188 molars) Group I (needleless): 94 teeth Group II (conventional needle): 94 teeth	A dose of 0.3 mL (3 units) of Ultracaine DS Forte™ was injected using a needle-free injection system.	A 27-gauge needle, measuring 50 mm in length and 0.40 mm in diameter, was used for injecting local anaesthesia.	Injection: 3.957 ± 2.616 Treatment: 2.913 ± 2.623 Post-treatment: 1.304 ± 1.987	Injection: 3.957 ± 3.252 Treatment: 2.087 ± 2.346 Post-treatment: 1.044 ± 1.873	-	-	A 27-gauge needle, measuring 50 mm in length and 0.40 mm in diameter, was used for injecting local anaesthesia.
5	Elbadry et al., 2023 [14]	Total sample: 38 children Group I (needleless): 18 children Group II (conventional needle): 18 children Mean age: 5.39 ± 0.86 years	The injection was done using a needleless jet injector with 4% benzocaine.	Buccal infiltration was performed using a conventional syringe with a 27-gauge short needle.	Wong-Baker FACES Pain Rating Scale: 0 (0-2); 0.526 ± 0.904	Wong-Baker FACES Pain Rating Scale: 8 (0-10); 7.68 ± 2.92	-	-	Buccal infiltration was performed using a conventional syringe with a 27-gauge short needle.
6	Oliveira et al., 2018 [15]	Total sample: 41 patients Males: 56.1% Females: 43.9% Mean age: 25.7 ± 4.4 years Group I (needleless): 41 teeth Group II (conventional needle): 41 teeth	2% lidocaine with 1:100,000 epinephrine was injected using a needleless jet injection method.	Buccal infiltration was performed using a conventional syringe with a 27-gauge short needle.	Visual Analogue Scale (VAS): 12.2 (0-55.4)	VAS: 12.1 (0-53.8)	Duration of pulpal anaesthesia: 20.0 minutes	Duration of pulpal anaesthesia: 40.0 minutes	Buccal infiltration was performed using a conventional syringe with a 27-gauge short needle.

Meta-analyses were carried out using RevMan version 5.4 (The Cochrane Collaboration, London, England, UK) with a random-effects model. The Q test was used to assess statistical heterogeneity, which was then quantified using the I^2^ statistic to evaluate the certainty of the evidence for each outcome. The following domains were considered: RoB, whereby each contributing study was appraised for potential methodological biases using a standardised RoB tool; consistency, in which the similarity of results across studies was examined to determine the level of heterogeneity; and publication bias, explored using funnel plots and statistical approaches, where applicable.

Results

**Figure 1:**
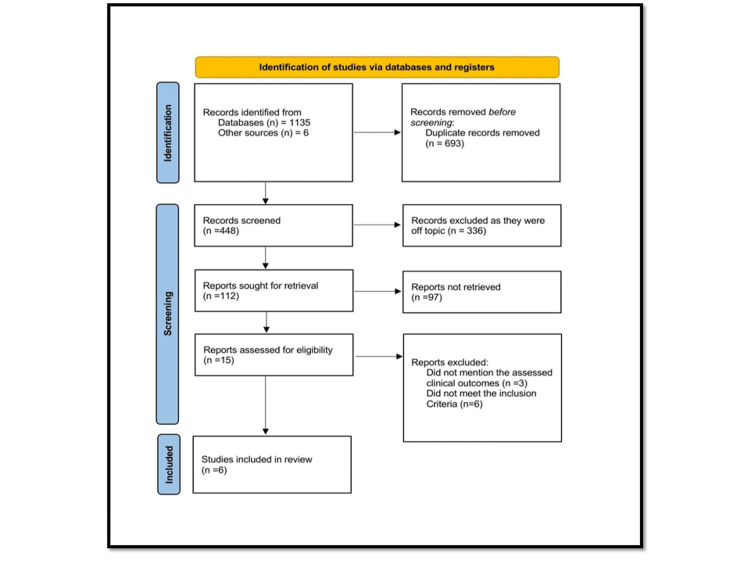
Flowchart representing the process of study selection.

An extensive electronic database search initially identified 1,135 articles. After eliminating duplicates, 448 unique records were retained. Titles and abstracts of 828 records were screened according to predefined eligibility criteria, leading to the exclusion of 433 articles deemed irrelevant. The full texts of 15 potentially eligible articles were retrieved and independently reviewed by two authors. After a detailed assessment against the eligibility criteria, six studies were found to meet all inclusion requirements and were included in the final analysis. The study selection process is illustrated in the PRISMA flow diagram (Figure 1).

Six studies investigated the effects of needleless jet injection versus conventional needle infiltration on pain perception and/or anxiety levels. Three studies [10-12] reported significantly lower pain levels with the needleless injection system during administration of LA. One study [11] also reported significantly lower anxiety during injection in the needleless group. Three studies [10,13,14] reported no significant difference in pain scores between the two groups, either during anaesthesia administration or the dental procedure.

Post-operative pain on the first day was assessed in two studies [12,13], with one [12] showing significantly lower pain in the needleless group, while the other [13] reported no difference. One study investigated local consequences, such as bleeding and haematoma, as well as anaesthesia success and onset time, and found no statistically significant differences between the two procedures [10]. All studies concluded that needleless jet injection is either equally effective or superior in terms of pain and acceptance compared to conventional needle-based infiltration in paediatric dental procedures.

**Figure 2:**
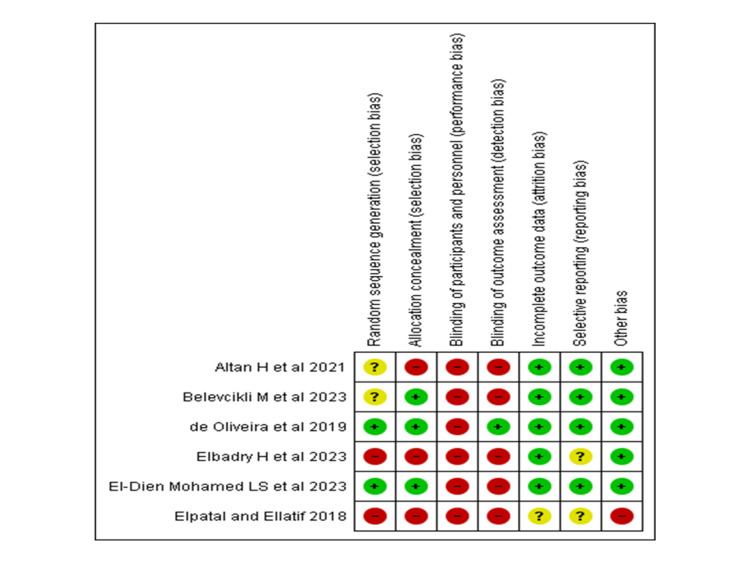
Risk of bias assessment of included randomized controlled trials. References: Altan et al. (2021) [11]; Belevickli et al. (2023) [13]; Oliveira et al. (2019) [15]; Elbadry et al. (2023) [14]; El-Dien Mohamed et al. (2023) [10]; Ellatif (2018) [12]

The RoB for the included RCTs was assessed using the Cochrane Collaboration’s tool (RoB 2). The method of random sequence generation was clearly reported in the studies by Oliveira et al. and El-Dien Mohamed et al. In contrast, Altan et al. and Belevcikli et al. mentioned that participants were randomised but did not provide details on the randomisation process. In the study by Elbadry et al., randomisation was conducted using two cards containing the names of the study groups, which is considered an inadequate method. Consequently, the random sequence generation domain was classified as having a high RoB (Figure 2).

Only three studies provided appropriate information on allocation concealment; hence, this domain was rated as having a high RoB. Due to the nature of the intervention, blinding both investigators and participants was not possible. Outcome assessor blinding was not generally documented, with the exception of Oliveira et al.'s study, in which the assessor was blind to group allocation. As a result, the overall RoB in this domain was assessed as high.

**Figure 3:**
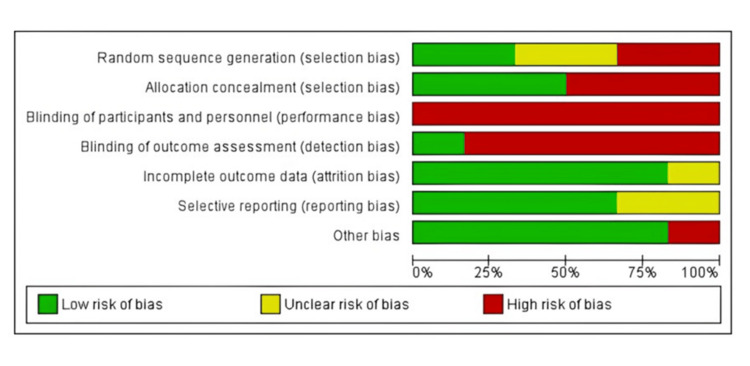
Summary of the overall risk of bias assessment for included randomized controlled trials.

Except for Ellatif's study, which was unclear about how many subjects were included in the final analysis, none of the studies revealed participant dropouts. Four studies had easily accessible study protocols and were thus classified as having a minimal RoB for selective reporting. Other potential causes of bias were identified due to a lack of information on sample size computation, inclusion and exclusion criteria, and examiner calibration. Except for Ellatif, all trials gave sample size estimates (Figure 3 and Table 3).

**Table 3:** Risk of bias assessment of included randomized controlled trials using the Cochrane RoB 2 tool.

Study	Random Sequence Generation (Selection Bias)	Allocation Concealment (Selection Bias)	Blinding of Participants and Personnel (Performance Bias)	Blinding of Outcome Assessment (Detection Bias)	Incomplete Outcome Data (Attrition Bias)	Selective Reporting (Reporting Bias)	Other Bias
Oliveira et al. [15]	Low risk: Computer-generated randomisation clearly described.	Low risk: Adequate concealment using sealed opaque envelopes.	High risk: Blinding not feasible due to the nature of the intervention.	Low risk: Outcome assessor explicitly blinded to group allocation.	Low risk: No dropouts; all participants analysed.	Low risk: Prespecified outcomes reported; protocol accessible.	Unclear risk: Examiner calibration not clearly reported.
El‑Dien Mohamed et al. [10]	Low risk: Random number tables used for sequence generation.	Low risk: Sequentially numbered opaque envelopes reported.	High risk: Blinding of participants and operators not possible.	Unclear risk: Assessor blinding not documented.	Low risk: No attrition reported.	Low risk: Outcomes reported as planned.	Unclear risk: Limited information on examiner calibration.
Altan et al. [11]	Unclear risk: Randomisation mentioned without methodological details.	High risk: Allocation concealment not reported.	High risk: No blinding possible or reported.	Unclear risk: Blinding of outcome assessor not specified.	Low risk: No participant dropouts reported.	Low risk: Prespecified outcomes reported.	Unclear risk: Inadequate reporting of inclusion/exclusion criteria and calibration.
Belevcikli et al. [13]	Unclear risk: Method of random sequence generation not described.	High risk: Allocation concealment not described.	High risk: Blinding not feasible due to intervention characteristics.	Unclear risk: Outcome assessor blinding not reported.	Low risk: Complete outcome data reported.	Low risk: Outcomes correspond to stated objectives.	Unclear risk: Sample size calculation and calibration details insufficient.
Elbadry et al. [14]	High risk: Inadequate randomisation using group-labelled cards.	High risk: No allocation concealment; predictable assignment.	High risk: No blinding of participants or personnel.	High risk: Outcome assessor blinding not reported.	Low risk: No attrition reported.	Low risk: Outcomes reported as described in methods.	Unclear risk: Potential operator-related bias; calibration not reported.
Ellatif [12]	Unclear risk: Insufficient details regarding random sequence generation.	High risk: Allocation concealment not reported.	High risk: Blinding not feasible due to intervention.	Unclear risk: Outcome assessor blinding not documented.	Unclear risk: Number of participants included in final analysis unclear.	Unclear risk: Study protocol not readily accessible.	Unclear risk: Sample size calculation and eligibility criteria insufficiently reported.

Assessment of Certainty of Evidence Using the Grading of Recommendations Assessment, Development and Evaluation (GRADE) Approach

GRADE assessment showed very low certainty evidence for pain during injection and low certainty evidence for pain during/after treatment. The certainty was downgraded due to high RoB related to lack of blinding, imprecision arising from small sample sizes and wide confidence intervals (CIs), and moderate heterogeneity for pain during injection. Publication bias could not be assessed due to the limited number of included studies. Therefore, the findings should be interpreted with caution (Table 4).

**Table 4:** GRADE summary of findings: INJEX versus conventional infiltration anaesthesia. ^1^ Risk of bias (downgraded one level): All included studies showed a high risk of performance bias due to the inability to blind participants and operators (needle vs. jet injector). Some studies also showed concerns in allocation concealment and detection bias, as demonstrated in the risk-of-bias summary. ^2^ Inconsistency (downgraded one level for pain during injection): Moderate statistical heterogeneity was observed (I^2^ = 64%), indicating variability in effect estimates across studies. ^3^ Imprecision (downgraded one level): Both outcomes had wide confidence intervals crossing the line of no effect, with a small total sample size, limiting precision and certainty of the pooled estimates. ^4^ Publication bias: Could not be assessed due to the inclusion of fewer than 10 studies, as per Cochrane and GRADE guidance. Downgrading was applied conservatively for pain during injection, where overall certainty was already very low. GRADE: Grading of Recommendations Assessment, Development and Evaluation; RCT: randomised controlled trial; SMD: standardised mean difference; CI: confidence interval

Outcome	No. of Studies (Participants)	Effect Estimate (SMD, 95% CI)	Risk of Bias	Inconsistency	Indirectness	Imprecision	Publication Bias	Certainty of Evidence (GRADE)
Pain during injection	2 RCTs (n = 244)	0.12 (-0.37 to 0.60)	Serious ↓^1^	Serious ↓^2^	Not serious	Serious ↓^3^	Not assessable ↓^4^	⊕◯◯◯ VERY LOW
Pain during/after treatment	2 RCTs (n = 244)	0.11 (-0.14 to 0.36)	Serious ↓^1^	Not serious	Not serious	Serious ↓^3^	Not assessable ↓^4^	⊕⊕◯◯ LOW

Meta-Analysis

Meta-analysis was conducted on two studies that provided sufficient quantitative data for analysis. The overall results are presented as a forest plot. Given that the heterogeneity among the included studies was substantial (I^2^ = 64%), a random-effects model was employed for the analysis.

**Figure 4:**
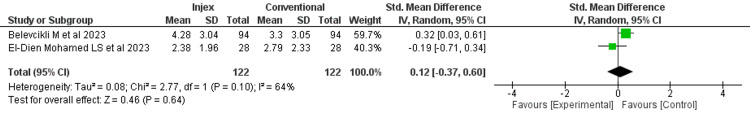
Forest plot comparing pain perception between needleless jet infiltration anaesthesia (INJEX) and conventional needle infiltration anaesthesia in children. References: Belevickli et al. (2023) [13] and El-Dien Mohamed et al. (2023) [10]

Comparison of pain during injection between needleless jet infiltration anaesthesia (using INJEX) and conventional infiltration anaesthesia: The pain in the needleless jet infiltration anaesthesia (using INJEX) group during injection did not differ significantly from that of the conventional infiltration anaesthesia group with a standardised mean difference of 0.12 (95% CI = -0.37 to -0.60; Z value = 0.46; p = 0.64) (Figure 4).

**Figure 5:**
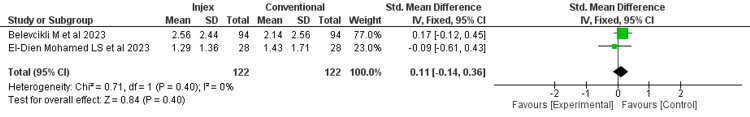
Comparison of pain during and after treatment between needleless jet infiltration anaesthesia (INJEX) and conventional infiltration anaesthesia. References: Belevickli et al. (2023) [13] and El-Dien Mohamed et al. (2023) [10]

Comparison of pain during/after treatment between needleless jet infiltration anaesthesia (using INJEX) and conventional infiltration anaesthesia: The pain in the needleless jet infiltration anaesthesia (using INJEX) group during/after treatment did not differ significantly from that of the conventional infiltration anaesthesia group with a standardised mean difference of 0.11 (95% CI = -0.14 to -0.36; Z value = 0.84; p = 0.40) (Figure 5).

Discussion

Pain management plays a central role in ensuring patient comfort and successful dental treatment. The introduction of local anaesthetics (LAs) has made dental procedures nearly painless. However, despite the effectiveness of LA, multiple needle insertions can cause high levels of apprehension among paediatric patients [10]. Needleless jet injection of LA has therefore emerged as a promising technique to control pain and reduce the fear of injections. Avoiding needles can lessen children’s dental fear and encourage a positive approach to dental treatment in the future.

El-Dien Mohamed et al. (2023) [10] found no significant difference in pain scores between needle-free and conventional anaesthesia groups. In contrast, Altan et al. (2021) [11] reported lower pain scores with the needle-free system, suggesting that the needle itself may act as a negative trigger for pain perception. Similarly, Ellatif (2018) [12] observed significantly lower pain scores on day one, measured using the FPS-R scale, in the needleless injection group compared to the conventional injection group.

Melek Belevcikli et al. (2023) [13] reported no significant differences in pain levels between needle-free injection systems and conventional dental needles during injection, treatment, and post-treatment phases for both filling and pulpotomy procedures.

In a study by Oliveira et al. (2018) [14], VAS scores revealed no significant difference in pain perception between the Comfort-In system and conventional dental injections in adults. Similarly, Satish Vishwanathaiah et al. (2024) [15] found that children in the INJEX group had significantly higher FBRS (Face, Behaviour, Recovery Scale) scores after anaesthesia administration than those receiving traditional injections, indicating less discomfort. The mechanism of needleless jet injections involves high-velocity delivery of anaesthetic solution through a narrow orifice, powered by either a spring or compressed gas, enabling tissue penetration without the need for a needle [15].

Currently, modern jet injection systems such as Madajet (Mada Medical Products, USA), INJEX, and Comfort-In (Mika Medical Co., South Korea) are being used in dentistry for LA administration [16-18]. However, only a limited number of studies have evaluated the effectiveness of these jet injectors during pulp therapy in children.

Conversely, Theocharidou et al. [19] reported that conventional infiltration anaesthesia was preferred over the needle-free Comfort-In system, with higher pain and discomfort observed during anaesthesia administration using the Comfort-In jet injector. In contrast, Ocak et al. [20] reported that the INJEX system caused less pain during injection compared with the conventional dental syringe.

The findings of the meta-analysis suggest that pain experienced with needleless jet infiltration anaesthesia (INJEX) is comparable to that reported with conventional infiltration anaesthesia during both injection and dental procedures. This could be attributed to the sudden sensation of pressure and the audible popping sound produced by the jet injector, which may trigger a fear response or cause patients to misinterpret the pressure as pain [8]. These findings are supported by Ocak et al. [20] and Yıldırım et al. [21], who reported that younger children may perceive the intensity of pain as higher than it actually is due to the noise and pressure sensation generated by the jet injection system.

Lautenbacher et al. [22] investigated pain levels during traditional dental anaesthesia and INJEX administration in 87 children and discovered that the jet injection technique was associated with more discomfort. The device's quick pressure and popping sound were thought to contribute to an increase in pain perception [8-10].

The present meta-analysis included only studies on infiltration anaesthesia of maxillary molars. Belevcikli et al. (2023) [13] reported no significant difference in pain intensity between maxillary and mandibular arches, with both Comfort-In and conventional methods providing effective anaesthesia, possibly due to the less dense bone in paediatric mandibles. Jet injectors remain limited to infiltration anaesthesia, unlike conventional techniques that support various delivery methods [23].

Several clinical trial studies reported adverse events associated with both methods. El-Dien Mohamed et al. (2023) reported that bleeding at the injection site was noted in 7.1% of the samples in the needleless jet injector group, while a higher percentage of 10.7% was noted in the conventional needle group. Other adverse effects, including haematoma at the injection site (10.7%) and bad taste (7.1%), were noted in the conventional needle group [10].

There are conflicting findings regarding the effectiveness of jet injection systems in achieving pulpal anaesthesia. Schoppink and Fernandez Rivas [7] found that the Madajet system could be successfully used for all clinical procedures involving primary teeth. In contrast, Dabarakis et al. [24] and Arapostathis et al. [8] found that the INJEX system was insufficient for providing adequate pulpal anaesthesia. Similarly, Brunton et al. [6] observed higher pain scores and an increased need for supplemental anaesthesia when using INJEX during dental extractions.

According to Albar et al. [25], jet injection enables faster administration of anaesthesia compared to conventional syringes. However, conventional needle-based anaesthesia was found to have a longer duration of action. The shorter effect of jet injectors limits their use in complex procedures. Despite a rapid onset (~1 ms), they may cause more pain during extractions due to the early fading of anaesthesia. Their limited anaesthetic volume, however, can be advantageous for patients with systemic risks [26].

A key limitation of jet injectors is their bulky design, which can hinder use in the paediatric oral cavity. Additionally, their appearance and the audible "pop" may induce fear in young patients [27]. Despite this, needleless systems offer several benefits: reduced pain and anxiety, rapid anaesthesia delivery, minimal head movement, and lower technique sensitivity compared to conventional needles.

This study has several limitations. Only six studies were included, with variations in sample characteristics, anaesthetic agents, and needle specifications. Most studies used a split-mouth design, where the first anaesthesia method may have influenced pain perception, especially in anxious children. Despite these factors, the inclusion of only RCTs strengthens the reliability and quality of the evidence. Also, the present meta-analysis included only two eligible RCTs, which inherently limits the statistical power and generalizability of the pooled estimates. Although a random-effects model was used in accordance with Cochrane recommendations to account for potential clinical and methodological heterogeneity, the small number of studies warrants cautious interpretation of the findings. The analysis reflects the current paucity of paediatric evidence on needle-free jet infiltration systems rather than a methodological limitation of the review itself. Publication bias assessment (e.g., funnel plot) was not performed because fewer than 10 studies were available, making such tests unreliable.

To conclude, needleless jet injection devices provide a novel method for delivering LA, helping to minimise fear and discomfort commonly associated with traditional needle-based techniques. Although the present meta-analysis did not find statistically significant differences in pain perception between the INJEX and conventional infiltration methods, the findings suggest that needleless jet injectors.

## Conclusions

The fear of LA administration is one of the key factors fueling anxiety in young children during dental procedures. Elimination of dental fear is very important and should be treated according to a patient-centred assessment. Proper delivery of LA can render dental treatments painless and comfortable for patients. Paediatric dentists face challenges in administering anaesthesia with minimal pain and discomfort.

Jet injection systems in dentistry represent an innovative and less intimidating method of delivering LA, aiming to alleviate fear associated with traditional injections. The results of the present meta-analysis indicate that the pain in the needleless jet infiltration anaesthesia (INJEX) group during injection and during treatment did not differ significantly from that of the conventional infiltration anaesthesia group. Needleless jet injection system procedure is a good alternative to the conventional needle approach and will always be more accepted with minimal postoperative pain by the children.
